# Assessing the sustainable development of a national research ecosystem: A generative AI-based evaluation of empirical educational research in China (2004–2023)

**DOI:** 10.1371/journal.pone.0341620

**Published:** 2026-01-28

**Authors:** Sen Wang, Yiming Wang

**Affiliations:** 1 Journal Center, East China Normal University, Shanghai, China; 2 Department of Radiology, Mayo Clinic, Rochester, Minnesota, United States of America; Universidad Especializada de las Americas, PANAMA

## Abstract

A nation’s progress toward Sustainable Development Goal 4 (Quality Education) depends in part on the long-term health of its educational research system, yet systematic, longitudinal assessments of such research ecosystems remain scarce. This study applies a generative artificial intelligence–based framework to evaluate the sustainable development of China’s empirical educational research ecosystem from 2004 to 2023. We compiled a dataset of 2,145 empirical studies published in leading Chinese education journals and used GPT-4o to score each paper on 31 quality indicators covering research problem, theoretical framing, design, data collection, analysis, and reporting, using a 1–10 analytic rating scale. Based on the resulting score distributions, we constructed a fuzzy relation matrix and applied a fuzzy comprehensive evaluation method to derive annual and overall sustainability indices, while the Criteria Importance Through Intercriteria Correlation (CRITIC) method was used to determine objective indicator weights. The overall sustainability index of China’s empirical educational research ecosystem over the 20-year period is 75.77 on a 100-point scale, with membership degrees concentrated at quality levels 7 (0.328) and 8 (0.435), indicating a generally robust and maturing system. Longitudinal trends reveal three stages of evolution—fluctuating development, rapid growth, and continuous improvement—corresponding to a shift toward more stable high-quality output. At the micro level, the ecosystem shows strong responsiveness to real-world educational problems, with high average scores for the relevance (8.45) and social significance (8.23) of research questions, as well as generally solid research design and data analysis practices. However, relatively lower scores for transparency of data analysis (7.08) and accessibility of raw data (6.46) highlight persistent challenges for reproducibility, open science, and methodological innovation. We conclude that China’s empirical educational research ecosystem has reached a relatively high and stable level of performance but faces critical tasks in strengthening data openness, methodological renewal, and AI-augmented governance. The proposed generative AI–based evaluation framework may offer a scalable tool for continuous monitoring and governance of national research ecosystems, while its results should be interpreted as an auxiliary input rather than a substitute for expert peer assessment.

## 1. Introduction

A nation’s capacity for sustainable social progress is inextricably linked to the vitality of its research and development systems. A robust, evidence-based educational research system is not merely an academic concern but a fundamental infrastructure for achieving the United Nations Sustainable Development Goal 4 (Quality Education). Building on UNESCO’s and OECD’s frameworks of research system sustainability, we define the sustainability of a national research ecosystem as its enduring capacity to generate socially relevant and methodologically robust knowledge, to maintain epistemic diversity and openness, and to adapt through feedback loops among research, policy, and practice in the face of evolving societal demands. As emphasized by global frameworks, the realization of inclusive and equitable quality education depends on continuous, data-driven innovation in educational practices and policies. High-quality empirical educational research acts as the cognitive engine for this endeavor, informing scientific decision-making and guiding effective reforms. However, assessing the long-term health, resilience, and evolutionary trajectory of such a vast academic ecosystem presents significant methodological challenges.

This study addresses this challenge by pioneering a novel methodology that assesses the sustainability of a national research ecosystem. It moves beyond traditional evaluation by using generative AI to conduct a data-simulated social experiment on two decades of empirical educational research from China (2004–2023). By shifting from merely “presenting results” to a “perspective of data simulation and application,” this research provides a macro-level analysis of the quality and dynamic trajectory of this vital academic field.

Drawing upon systems theory and models of research system sustainability advocated by organizations such as UNESCO and the OECD, we conceptualize a national research ecosystem as a complex adaptive system. In this context, “sustainability” extends beyond the mere accumulation of outputs. It refers to the system’s capacity for self-organization, continuous knowledge regeneration, and resilience in response to changing societal demands. More specifically, we draw on diffusion-of-innovation perspectives to understand how methodological advances spread across journals and subfields, and on research evaluation scholarship that treats publication systems as complex, adaptive networks. This theoretical grounding allows us to interpret the empirical trends identified by the GAI-based assessment as manifestations of deeper system-level dynamics rather than isolated quality signals. A sustainable research ecosystem is characterized by a dynamic equilibrium where methodological innovation diffuses effectively, and research paradigms evolve to address emerging real-world complexities. Understanding this evolutionary mechanism is crucial for academic governance.

By focusing on China as a rapidly developing major academic system, this study contributes to global debates on how national research ecosystems can be steered toward SDG 4. Specifically, our GAI-based evaluation sheds light on whether and how empirical educational research can simultaneously respond to pressing policy agendas, uphold methodological rigor, and build long-term capacities for innovation and international knowledge exchange. These questions are central not only to China but also to other countries seeking to make their research systems more sustainable, equitable, and globally connected. The significance of this study lies in its contribution to a more sustainable model of academic assessment and governance. First, by analyzing a “twenty-year” timespan, it establishes an evolvable evaluation baseline, creating a platform for longitudinal monitoring of a research ecosystem’s health and resilience. Second, the AI-driven simulation offers data-driven insights that can help researchers identify shortcomings and support policymakers in formulating strategies for sustainable academic innovation and educational reform. Third, it responds to the need for more efficient and intelligent academic evaluation, providing a replicable, AI-based framework that can enhance the precision of research assessments and guide the high-quality, sustainable development of scholarship.

### 1.1. The evolution and sustainability of research paradigms in education

The long-term sustainability of any academic field is heavily influenced by the evolution and dominance of its research paradigms. Within the global educational research community, the empirical paradigm, which prioritizes quantitative analysis and causal inference, has become the predominant methodological framework, particularly in the Anglophone world. Driven by advances in statistical theory and standardized testing, this paradigm shifted educational research toward a framework emphasizing objective data and evidence-based conclusions [1,2]. This methodological orientation has been disseminated globally through academic publishing networks and international collaborations, shaping research practices worldwide. The extent of this shift is evident in scientometric analyses. For example, the proportion of empirical studies in Germany’s PSYNDEX educational psychology database rose from 48% in the 1979–1987 period to 73.6% in subsequent years [3].

Despite its widespread influence, the adoption of this empirical paradigm is not monolithic, and its application varies across different niches of the research ecosystem. Journals focused on specific domains like higher education often show a high proportion of empirical articles, whereas those in teacher education may favor mixed-methods approaches that blend quantitative data with qualitative interviews. Meanwhile, comprehensive journals like the *American Educational Research Journal* attempt to balance theoretical and empirical contributions, though the share of empirical work has steadily increased [4,5]. This heterogeneity suggests a degree of adaptability within the broader system; however, the fundamental trend toward a quantitative, evidence-based paradigm continues to expand its global influence.

Against this backdrop of global paradigmatic evolution, understanding the trajectory of empirical research in China has become a critical task for assessing the sustainable development of its national research ecosystem. While China has made progress in this area, its research ecosystem faces unique sustainability challenges. Systematic studies analyzing its historical development, operational models, and pathways to innovation remain limited. A significant concern for the long-term health of this ecosystem is the relative lack of visibility of Chinese empirical educational research in mainstream international journals. This limited integration into global academic dialog not only hinders the international community’s understanding of China’s educational practices but also curtails the potential contributions of Chinese scholarship to global knowledge. Such isolation poses a risk to the ecosystem’s sustainability, as robust, long-term development depends on reciprocal knowledge exchange and international collaboration. Therefore, an in-depth assessment of the quality and developmental dynamics of empirical educational research in China is urgently needed to foster its high-quality, sustainable growth and integration into the global scientific community providing a replicable, AI-based framework that can enhance the precision of research assessments. By validating this model within the context of China—a rapidly developing major academic power—this study contributes to the international discourse on research evaluation, offering insights applicable to other nations striving to align their educational research systems with global sustainability targets.

### 1.2. Data simulation as a tool for sustainable research intelligence

The pursuit of a sustainable research ecosystem necessitates the adoption of innovative technologies that can enhance efficiency, foresight, and adaptability. Data simulation has emerged as a particularly powerful technology in educational research, offering significant technical advantages that drive the field’s transformation toward data-intelligent models. By leveraging computational and mathematical methods to mimic the behavior of real-world systems, data simulation generates artificial datasets that adhere to specific statistical properties or logical rules. The core purpose of this technology is to supplement or replace real data in various contexts, including theory validation, algorithm testing, and system optimization, thereby offering advantages in cost, time, flexibility, and risk mitigation [6–10].

The application of data simulation across multiple scenarios is critical for building a more resilient and forward-looking educational research system. For instance, in policy evaluation, simulation allows for the predictive quantification of policy impacts by modeling variables like resource allocation and population shifts, thus bypassing the costs and limitations of traditional pilot studies [11]. In optimizing teaching systems, dynamic models informed by learning behavior data can support personalized learning pathways, helping to identify knowledge gaps and refine instructional strategies for greater efficacy [12]. Furthermore, simulation provides safe, risk-free environments for teacher training, allowing educators to practice classroom management strategies while enabling the extraction of effective teaching models from behavioral data [13]. It also aids in teaching complex concepts by transforming abstract theories into interactive, visual models that promote interdisciplinary thinking and knowledge integration [14].

The technical strengths of data simulation—including parameterized modeling that transcends spatiotemporal constraints, dynamic simulation of non-linear processes, and safe virtual spaces for trial and error—collectively enhance the generalizability and robustness of research conclusions. By providing verifiable quantitative evidence at a lower cost and risk, data simulation propels educational research toward a more data-driven, intelligent framework, continuously empowering the scientification of education and contributing to the long-term sustainability of the research ecosystem.

### 1.3. Generative artificial intelligence and the sustainability of academic evaluation

The sustainability of a research ecosystem is fundamentally dependent on the integrity and efficiency of its quality control mechanisms, with academic evaluation serving as the cornerstone. However, the conventional academic evaluation process—encompassing submission, screening, review, and decision-making—is often fraught with inefficiencies that threaten its long-term viability. Persistent challenges such as protracted review cycles, reviewer subjectivity and bias, the scarcity of qualified experts, and difficulties in detecting methodological or ethical flaws can create significant bottlenecks, strain resources, and hinder the timely dissemination of knowledge [15–17]. Addressing these issues is crucial for building a more resilient and sustainable model of academic governance.

Generative Artificial Intelligence (GAI), powered by sophisticated deep learning models like GANs, VAEs, and transformer architectures, offers a pathway to transforming this traditional model, enhancing both its efficiency and robustness [18–20]. GAI technologies can be strategically deployed to alleviate critical pressures at various stages of the review process. During initial manuscript screening, GAI can automate laborious tasks like format compliance, plagiarism checks, and the identification of machine-generated text [21]. For substantive content review, it can assist human experts by generating concise summaries of core arguments, correcting linguistic errors, and offering preliminary evaluations of a study’s novelty and potential contributions [22,23].

Furthermore, GAI can bolster the sustainability of the review workflow by producing structured feedback, optimizing reviewer assignment through topic modeling, and even predicting manuscript acceptance probabilities based on an aggregation of review outcomes [24,25]. Despite this transformative potential, the responsible and sustainable integration of GAI requires navigating significant challenges, including the risk of content hallucination, algorithmic bias, data privacy concerns, and unresolved issues of accountability [26,27,28]. Nevertheless, overcoming these hurdles is essential, as the continued development of GAI-augmented systems promises a more efficient, reliable, and ultimately sustainable future for academic evaluation.

Therefore, to address the identified gaps and challenges, this study pursues four primary aims. First, we develop and validate a novel methodology that utilizes generative artificial intelligence for the large-scale, longitudinal assessment of a national research ecosystem’s quality and sustainability. Second, we apply this framework to conduct a comprehensive evaluation of China’s empirical educational research from 2004 to 2023, generating a data-driven profile of its development over two decades. Third, based on this evaluation, we identify the macro-level evolutionary phases of the ecosystem and diagnose its specific micro-level strengths and vulnerabilities that impact its long-term health. Finally, we formulate a set of targeted, evidence-based recommendations designed to enhance the ecosystem’s resilience, innovative capacity, and sustainable integration into the global academic community.

## 2. Materials and methods

### 2.1. Conceptual framework for a sustainable GAI-based evaluation

The sustainable development of a national research ecosystem requires objective, efficient, and scalable methods for quality assessment. With the widespread adoption of GAI, its remarkable capabilities in natural language processing and text generation offer a novel solution for enhancing the evaluation of empirical educational research [29–32]. This study operationalizes this potential by utilizing a large language model as a data simulation tool, treating its generated analyses as the primary research data. This approach is grounded in existing research demonstrating that AI can maintain a high degree of objectivity and fairness in academic evaluation tasks, thereby reducing the subjective biases inherent in manual assessments [33]. Moreover, GAI represents a promising pathway for resolving persistent challenges in traditional academic evaluation, and models like GPT-4 have been shown to produce feedback that significantly overlaps with that of human experts [34], with performance being further enhanced through optimized prompts and model selection [35].

To construct a robust and sustainable instrument for this ecosystem-level assessment, this study utilized “GPT-4o” (OpenAI) as the primary data simulation tool, with formal data collection occurring from 26 May to 15 June 2024. To ensure reproducibility and minimize the stochastic nature of GAI outputs, AI models were configured with default parameters. The specific prompt structure, designed through iterative engineering, adopted a structured, stepwise, rubric-based prompting strategy aligned with the 31 analytic indicators, explicitly instructing the model to (1) read the full text of the paper, (2) review the 31 quality indicators, (3) extract key evidence relevant to each indicator, and (4) assign a score from 1 to 10 for each indicator based on the rubric.

Prior to the full-scale evaluation, a crucial pilot test was conducted on 131 empirical educational research papers of *Journal E between 2013 and 2023*. The purpose of this preliminary phase was threefold: (a) to verify the stability and consistency of the GAI tool for reliable longitudinal analysis; (b) to assess its consistency relative to other leading models, namely Gemini 1.5 Pro (Google) and Qwen 2.5 (Alibaba Cloud Tongyi Qianwen); and (c) to establish the validity of the GAI-generated scores as a credible measure of research quality. To ensure comparability, Gemini 1.5 Pro and Qwen 2.5 were configured to mirror the GPT-4o setup as closely as their respective platforms allowed. All models were used the same system role and stepwise evaluation instruction. These models were not used for the main corpus evaluation but solely for cross-model reliability testing.

### 2.2. Establishing methodological rigor: Validation of the GAI evaluator

#### 2.2.1. Stability validation.

**Fig 1:**
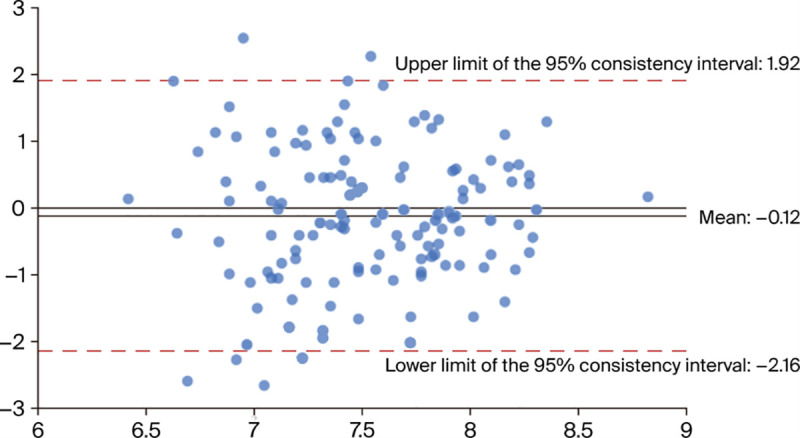
Bland–Altman analysis of two assessments.

The capacity for longitudinal analysis is central to assessing sustainable development. Therefore, the stability of the evaluation tool is paramount. To assess whether GPT-4o provides consistent results over time, this study employed the Bland–Altman analysis method to compare two separate pilot tests. The analysis revealed a mean difference between the two tests of −0.12, which is close to zero, indicating the absence of systematic bias. With only 0.046% (6/131) of data points falling outside the 95% limits of agreement (LoA), the consistency was high. The 95% confidence interval of the mean difference was [−0.299, 0.058], which contains zero, further confirming that there were no significant differences between the two tests (*p* = 0.906). The narrow LoA range of [−2.162, 1.921] and a coefficient of repeatability of 2.048 demonstrated that measurement differences were within an acceptable range and that the tool’s repeatability was good, making it suitable for a long-term study (Fig 1).

#### 2.2.2. Reliability validation.

A sustainable evaluation system should not be entirely dependent on a single proprietary tool. To measure the consistency of evaluations generated by multiple AI models, this study conducted a Kendall’s W consistency test using scores obtained from GPT-4o, Gemini 1.5 Pro, and Qwen 2.5. The results yielded a Kendall’s W coefficient of 0.441, with a chi-square value of 115.461 and a *p*-value of 0.000, indicating a statistically significant, non-random convergence among the models. While differences in scoring patterns were observed—with Gemini 1.5 Pro being most concentrated and Qwen 2.5 showing the greatest variability—the overall findings reflected “moderate consistency”, confirming that the evaluation pattern is a generalizable characteristic of GAI and not an artifact of a single model (Table 1).

**Table 1:** Analysis of different generative artificial intelligence tools using Kendall’s W.

Name	Rank Average	Median
GPT-4o	2.042	7.548
Gemini 1.5 pro	1.317	6.71
Qwen 2.5 (Trial 1)	2.641	8.258

#### 2.2.3. Validity analysis.

For a GAI-based assessment to be a meaningful tool for ecosystem evaluation, its outputs must align with established benchmarks of academic quality. To systematically assess this, the study performed a validity analysis by inviting multiple domain experts to manually score a sample of papers. The expert panel consisted of five professors from leading universities, each with over 10 years of experience in educational research and editorial service. Their expertise covered educational psychology, curriculum and instruction, higher education studies, and educational assessment, and the panel included both male and female scholars. To assess inter-rater reliability among the human experts prior to comparison with the AI, a Kendall’s W test was conducted, yielding a coefficient of 0.78 (p < 0.01), indicating strong consensus on the ground truth quality of the sampled papers. A Spearman correlation analysis, performed on the expert scores and the AI evaluations, produced a correlation coefficient of 0.921, which was significant at the 1% level, indicating a high positive correlation. The validity of the AI tool was further supported through cross-validation against traditional bibliometric indicators, namely “citation frequency” and “download frequency”, confirming its utility as a credible proxy for measuring the quality of empirical papers.

### 2.3. Data corpus and evaluation protocol

#### 2.3.1. Sample selection and corpus construction.

To conduct a meaningful assessment of a national research ecosystem’s sustainable development, it is essential to curate a representative corpus of its core intellectual output. Academic journal articles serve as the primary indicators of the developmental trajectory and quality of a research field. Therefore, a critical methodological challenge is to select journals whose publications accurately reflect the state of high-level empirical educational research in China over the two-decade period.

This study addresses this challenge through purposive judgmental sampling, choosing journals based on a comprehensive set of criteria designed to ensure the sample mirrors the stability, diversity, and quality of the national research landscape. These criteria included the proportion of empirical articles published, disciplinary breadth, the nature of sponsoring institutions (e.g., research institutes, “Double First-Class” universities), publication quality (as indicated by Chinese Social Sciences Citation Index [CSSCI], which is a prestigious citation index system in China. It is developed and maintained by the Institute for Social Sciences Information of Nanjing University, aiming to evaluate and index high-quality academic journals in the field of social sciences.), geographical distribution (covering north regions, south regions and east–central–west regions), and publication longevity (a history exceeding two decades). Based on this multi-faceted approach, four leading CSSCI-indexed educational journals were selected: *Journal A, Journal B, Journal C, Journal D*. The final corpus includes all empirical papers published in these journals between 2004 and 2023.

In operational terms, the corpus was constructed through a three-stage filtering process. First, we retrieved all records published between 2004 and 2023 in the four selected CSSCI-indexed educational journals from the CNKI database, including research articles, editorials, book reviews, conference reports, and other non-research content. Second, non-academic items such as editorial notes, news reports, book reviews, and calls for papers were manually excluded to retain only full-length research articles. Third, we applied the predefined operational definition of empirical educational research—emphasizing objectivity, evidence-based conclusions, and testability in quantitative, qualitative, or mixed-methods designs—to identify empirical studies. This stepwise procedure ensures that the corpus accurately reflects the core empirical output of China’s educational research community over the two-decade period.

#### 2.3.2. Evaluation framework for ecosystem sustainability.

To systematically assess the sustainable development of a national research ecosystem, a robust and multidimensional evaluation framework is required. This study adapts the Evaluation Framework for Empirical Research Papers, an instrument developed and refined by the Editorial Department of the *Journal B* This foundational framework was selected for its demonstrated reliability, having been established through rigorous literature review, extensive empirical testing, and iterative validation incorporating feedback from over 300 experts across diverse disciplines within China’s educational research community. To tailor this tool for a macro-level ecosystem analysis, several modifications were made to align with the study’s objectives.

First, indicators related to academic ethics and misconduct were excluded from the evaluation. For a longitudinal assessment of a research ecosystem’s published output, compliance with fundamental academic norms is considered a baseline prerequisite for a paper’s inclusion in the scholarly record. The selected journals have their own academic evaluation and editorial processes to screen for such issues before publication. Therefore, this study’s focus shifted from re-evaluating gatekeeping functions to assessing the substantive academic quality of the research that has successfully entered the ecosystem.

Second, to directly measure the innovative capacity and long-term impact crucial for a sustainable research ecosystem, the framework was augmented with indicators assessing a paper’s originality and contribution. Specifically, metrics evaluating the “sustained influence of the research topic”, “contribution to the disciplinary knowledge system”, and “contribution to the broader knowledge system” were incorporated. While these dimensions traditionally involve a high degree of subjective judgment, this study leverages the advanced capabilities of generative AI. By analyzing a vast textual corpus and integrating interdisciplinary knowledge, AI can provide a preliminary, data-simulated evaluation of a paper’s potential impact, offering a scalable method to assess an ecosystem’s innovative health, even while acknowledging that such results are an auxiliary tool and not a substitute for the academic community’s long-term cognitive consensus.

Finally, to ensure the granular and precise diagnosis of the ecosystem’s performance, this study employs the framework’s secondary indicators directly. These specific metrics, which underpin primary dimensions, like research innovativeness, design, process, and outcomes, offer more operable and detailed measurement standards. This approach allows for a comprehensive and nuanced assessment, ensuring that critical aspects of research quality are not overlooked and enabling the identification of specific strengths and weaknesses within the ecosystem, thereby providing targeted insights for its continuous and sustainable improvement.

#### 2.3.3. A profile of quality in the research ecosystem.

This study conducted a comprehensive evaluation of 2145 empirical educational research papers published between 2004 and 2023, utilizing the generative AI tool GPT-4o to score each paper against 31 distinct quality indicators on a 10-point scale. All 31 indicators were operationalized as analytic rating scales ranging from 1 to 10, where 1 represents “very weak” performance and 10 indicates “excellent” performance relative to the standards of high-quality empirical educational research. For each indicator, the evaluation rubric combined (a) a concise conceptual definition and (b) concrete scoring anchors describing typical features at low (1–3), medium (4–7), and high (8–10) levels. The results provide a multi-faceted diagnosis of the Chinese empirical educational research ecosystem, revealing its core characteristics, developmental trajectory, and overall sustainability (Table 2).

**Table 2:** Descriptive statistics of gai-generated quality scores for Chinese empirical educational research papers (2004–2023).

Indicator	Minimum Value	Maximum Value	Average Value	Standard Deviation	Median
Sustained Impact of Research Topics	5	10	7.76	0.76	8
Novelty of Research Methods	5	10	7.27	0.89	7
Contribution to the Discipline Knowledge System	5	10	7.63	0.77	8
Contribution to the Overall Knowledge System	4	10	7.03	0.80	7
Specificity of Problem Description	5	10	8.19	0.73	8
Relevance of Research Problem	5	10	8.45	0.74	9
Appropriateness of Research Design	5	10	7.61	0.69	8
Rationality of Research Design	5	10	7.56	0.70	8
Alignment of Methods with Research Objectives	5	10	7.83	0.74	8
Robustness of Methodology	4	9	7.27	0.76	7
Relevance of Theory to Research Problem	5	10	7.89	0.71	8
Consistency between Theory and Hypotheses	4	9	7.55	0.75	8
Explanatory Power of Theory	4	10	7.49	0.74	8
Appropriateness of Data Analysis Methods	5	10	7.63	0.85	8
Reliability of Data Analysis Results	5	9	7.50	0.79	8
Transparency of Data Analysis	4	9	7.08	0.84	7
Academic Significance	5	10	7.91	0.74	8
Social Significance	5	10	8.23	0.91	8
Coherence of Content	6	10	7.87	0.68	8
Consistency between Content and Conclusion	5	10	7.84	0.71	8
Sufficiency of Argumentation	5	10	7.53	0.73	8
Rationality of Argumentation	5	10	7.64	0.70	8
Matching of Data Presentation with Original Data	5	9	7.56	0.82	8
Correspondence between Data and Text Descriptions	5	10	7.56	0.79	8
Internal Consistency of Research Findings	5	9	7.63	0.71	8
Detail of Research Methods and Data Collection	4	10	7.36	0.81	7
Availability of Original Data	3	9	6.46	0.86	6
Verification of Result Stability	4	10	7.12	0.78	7
Direct Correspondence between Conclusions and Results	5	9	7.79	0.71	8
Consistency between Data Analysis and Conclusions	5	10	7.75	0.72	8
Explanation of Research Limitations	4	9	6.86	0.71	7

On a micro-level, the GAI-generated scores for most indicators were highly concentrated around an average of 7, with standard deviations generally between 0.7 and 0.9. This suggests a consistent application of research standards across the ecosystem’s published output. The analysis highlights several key strengths that characterize the research landscape. First, there is a profound focus on real-world issues, evidenced by the highest average scores in the “relevance of research problem” (8.45) and its “social significance” (8.23). This indicates a research ecosystem that is highly responsive to practical educational challenges and societal demands. Second, the ecosystem prioritizes rigorous research design and the integration of theory with practice, with strong scores in indicator sets related to these areas (ranging from 7.55 to 7.89). Finally, a commitment to rigorous data analysis and processing is evident from consistently high scores in related metrics.

However, the evaluation also identified critical areas for improvement necessary for enhancing the ecosystem’s long-term sustainability and global competitiveness. Specifically, indicators for “transparency of data analysis” (7.08) and “accessibility of raw data” (6.46) received lower scores. This points to a systemic challenge in data sharing and transparency, which limits the reproducibility of studies and hinders broader academic discourse.

## 3. Results

### 3.1. Systematic evaluation based on the fuzzy comprehensive evaluation method and the CRITIC method

The fuzzy comprehensive evaluation method is adopted because research quality and ecosystem sustainability involve inherently fuzzy boundaries, and the system-level state emerges from numerous partially overlapping dimensions. Fuzzy sets provide a natural way to aggregate such graded membership relationships into an interpretable overall sustainability score. The fuzzy relation matrix provides a comprehensive profile of the ecosystem’s performance, disaggregated across all 31 quality dimensions. However, to synthesize these multi-dimensional data into a single, meaningful measure reflective of the ecosystem’s overall health and sustainability, the relative importance of each indicator must be established. A simple, unweighted aggregation would incorrectly assume that all facets of research quality contribute equally to the system’s long-term resilience and developmental capacity. To construct a valid and objective assessment, it is therefore necessary to assign a weight to each indicator that reflects its unique contribution. To move beyond subjective expert judgments, this study utilizes the Criteria Importance Through Intercriteria Correlation (CRITIC) method to determine these weights objectively. The CRITIC method assigns weights based on the contrast intensity and conflict within the data. In the context of ecosystem sustainability, indicators with higher variability represent active, evolving frontiers of the research system, while indicators with low variability typically signify standardized, mature “hygiene” factors. CRITIC allows the evaluation to dynamically reflect the ecosystem’s evolutionary drivers rather than static standards. This makes CRITIC particularly suitable for sustainability-oriented assessments of research ecosystems, where the goal is not merely to reward uniformly high scores but to identify which dimensions are actively driving systemic transformation.

#### 3.1.1. Construction of the fuzzy relation matrix.

To translate the vast dataset of 2145 individual paper evaluations into a cohesive, system-level assessment, the first step is to construct a fuzzy relation matrix. This matrix quantitatively maps the overall performance of the research ecosystem against the established quality criteria (Table 3). The process begins by defining the evaluation set, which consists of the 31 quality indicators and a 10-level scoring rubric representing performance from poor (1) to excellent (10).

**Table 3:** Frequency distribution of scores across 31 quality indicators (N = 2,145).

Indicator	Scores									
	1	2	3	4	5	6	7	8	9	10
Sustained Impact of Research Topics	0	0	0	0	8	102	584	1163	284	4
Novelty of Research Methods	0	0	0	0	33	339	987	598	185	3
Contribution to the Discipline Knowledge System	0	0	0	0	9	151	652	1141	187	5
Contribution to the Overall Knowledge System	0	0	0	3	43	442	1106	497	52	2
Specificity of Problem Description	0	0	0	0	1	35	290	1053	758	8
Relevance of Research Problem	0	0	0	0	1	15	186	843	1012	88
Appropriateness of Research Design	0	0	0	0	2	108	758	1143	133	1
Rationality of Research Design	0	0	0	0	3	140	769	1120	112	1
Alignment of Methods with Research Objectives	0	0	0	0	1	86	542	1176	335	5
Robustness of Methodology	0	0	0	1	18	285	1005	770	66	0
Relevance of Theory to Research Problem	0	0	0	0	2	61	476	1246	356	4
Consistency between Theory and Hypotheses	0	0	0	1	8	164	768	1057	147	0
Explanatory Power of Theory	0	0	0	1	7	171	847	994	124	1
Appropriateness of Data Analysis Methods	0	0	0	0	16	178	681	976	291	3
Reliability of Data Analysis Results	0	0	0	0	15	204	774	996	156	0
Transparency of Data Analysis	0	0	0	4	57	415	1014	592	63	0
Academic Significance	0	0	0	0	4	64	470	1206	387	14
Social Significance	0	0	0	0	4	85	332	789	856	79
Coherence of Content	0	0	0	0	0	58	473	1303	310	1
Consistency between Content and Conclusion	0	0	0	0	3	66	518	1243	314	1
Sufficiency of Argumentation	0	0	0	0	7	157	806	1045	129	1
Rationality of Argumentation	0	0	0	0	5	117	672	1211	139	1
Matching of Data Presentation with Original Data	0	0	0	0	16	192	720	1000	217	0
Correspondence between Data and Text Descriptions	0	0	0	0	18	177	706	1075	168	1
Internal Consistency of Research Findings	0	0	0	0	4	126	678	1182	155	0
Detail of Research Methods and Data Collection	0	0	0	1	21	264	904	840	114	1
Availability of Original Data	0	0	3	18	231	832	869	180	12	0
Verification of Result Stability	0	0	0	2	48	353	1078	626	37	1
Direct Correspondence between Conclusions and Results	0	0	0	0	4	88	529	1258	266	0
Consistency between Data Analysis and Conclusions	0	0	0	0	3	103	555	1245	238	1
Explanation of Research Limitations	0	0	0	4	53	531	1222	330	5	0

For each of the 31 indicators, the frequency distributions of scores $f_{ij}$ across the 10 performance levels were calculated based on the entire sample of papers. This captures the ecosystem’s aggregate performance on each specific dimension of quality. To create a standardized basis for comparison, these raw frequencies were then normalized to derive the fuzzy frequencies $r_{ij}$ This normalization organizes the performance data into a comprehensive fuzzy relation matrix, R, which represents the degree of membership for each indicator at each quality level.

The matrix R is constructed as follows.

First, the total frequency $S_{i}$ for each indicator is calculated:


$$S_{i}=\sum_{j=\text{1}}^{\text{1}\text{0}}f_{ij}$$ (1)

Next, each frequency $f_{ij}$ is normalized to obtain the fuzzy frequency $r_{ij}$, and these are organized into the 31 × 10 fuzzy relation matrix R:


$$R=(\begin{array}{cccc} r_{\text{1}\text{1}} & r_{\text{1}\text{2}} & \cdots & r_{\text{1},\text{1}\text{0}}\\ r_{\text{2}\text{1}} & r_{\text{2}\text{2}} & \cdots & r_{\text{2},\text{1}\text{0}}\\ \vdots & \vdots & \ddots & \vdots \\ r_{\text{3}\text{1},\text{1}} & r_{\text{3}\text{1},\text{2}} & \cdots & r_{\text{3}\text{1},\text{1}\text{0}} \end{array})$$ (2)

where $r_{ij}$ represents the normalized frequency of indicator i at score level j. This matrix provides a detailed and structured representation of the ecosystem’s quality profile, serving as the essential input for the subsequent comprehensive evaluation.

#### 3.1.2. Determination of weight vector using the CRITIC method.

To move from a descriptive profile to a synthetic evaluation of the ecosystem’s sustainability, the relative importance of each quality indicator must be determined. Not all 31 indicators contribute equally to the long-term vitality and resilience of the research landscape; therefore, an objective weighting system is required to ensure the final assessment accurately reflects the ecosystem’s structural priorities.

This study employs the CRITIC method to objectively calculate these weights. The primary strength of this method is that it derives weights directly from the inherent statistical properties of the data, thereby avoiding the subjectivity of expert-assigned scores and revealing the de facto priorities that govern the ecosystem’s development. The method’s logic is grounded in two key principles directly relevant to a sustainability assessment: (1) Measured by the standard deviation, this reflects the degree of performance variation across the research sample. An indicator with higher variability signifies greater contrast and thus contains more information for differentiating quality levels within the ecosystem, highlighting areas of inconsistency that are critical for targeted improvements. (2) Assessed through the correlation matrix, this quantifies the uniqueness of the information provided by each criterion. Indicators that have a low correlation (high conflict) with others represent distinct, non-redundant dimensions of research quality, making it vital for a holistic and robust assessment of the ecosystem’s multi-faceted health.

The CRITIC method integrates these two aspects to quantify the total information content ($I_{j}$) of each indicator. The specific formula is as follows:


$$I_{j}=s_{j}\cdot \left(\sum_{k\neq j}\left|\text{1}-r_{jk}\right|\right)$$ (3)

where $s_{j}$ is the standard deviation of the *j-th* indicator and $r_{jk}$ is the correlation coefficient between the *j-th* and *k-th* indicators.

Finally, the objective weight for each indicator ($W_{j}$) is determined by normalizing its information content relative to the total information across all indicators, as follows:


$$W_{j}=\frac{I_{j}}{\sum_{j=\text{1}}^{n}I_{j}}$$ (4)

This process yielded a weight vector W for the 31 indicators based on the analysis of 2145 papers, providing an objective foundation for the subsequent comprehensive evaluation.


$$\text{W}=\left({\text{w}}_{\text{1}},{\text{w}}_{\text{2}},\ldots ,{\text{w}}_{\text{31}}\right)$$ (5)

The indicator weights derived from the CRITIC method (Table 4) reveal the de facto structural priorities that have shaped the sustainability of China’s empirical educational research ecosystem over the last two decades. An analysis of these weights highlights the key drivers of the ecosystem’s health, resilience, and adaptive capacity.

**Table 4:** Objectively derived weights for the 31 quality indicators using the CRITIC method.

Indicator	Indicator Variability	Indicator Conflict	Information Volume	Weight
Social Significance	0.907	16.67	15.118	5.34%
Novelty of Research Methods	0.886	13.711	12.149	4.29%
Availability of Original Data	0.864	13.788	11.914	4.21%
Specificity of Problem Description	0.732	15.644	11.459	4.05%
Relevance of Research Problem	0.742	15.039	11.163	3.94%
Contribution to the Overall Knowledge System	0.797	13.06	10.409	3.68%
Transparency of Data Analysis	0.839	11.901	9.982	3.53%
Contribution to the Discipline Knowledge System	0.766	12.623	9.667	3.42%
Appropriateness of Data Analysis Methods	0.85	11.343	9.646	3.41%
Matching of Data Presentation with Original Data	0.82	11.771	9.652	3.41%
Detail of Research Methods and Data Collection	0.806	11.796	9.502	3.36%
Explanatory Power of Theory	0.744	12.697	9.444	3.34%
Correspondence between Data and Text Descriptions	0.788	11.681	9.209	3.25%
Sustained Impact of Research Topics	0.76	11.916	9.055	3.20%
Consistency between Theory and Hypotheses	0.752	11.922	8.971	3.17%
Explanation of Research Limitations	0.711	12.212	8.686	3.07%
Robustness of Methodology	0.762	11.352	8.648	3.06%
Reliability of Data Analysis Results	0.792	10.808	8.564	3.03%
Academic Significance	0.744	11.517	8.571	3.03%
Relevance of Theory to Research Problem	0.711	11.973	8.51	3.01%
Alignment of Methods with Research Objectives	0.744	11.091	8.247	2.91%
Coherence of Content	0.677	12.032	8.148	2.88%
Verification of Result Stability	0.784	10.406	8.157	2.88%
Appropriateness of Research Design	0.687	11.384	7.822	2.76%
Rationality of Research Design	0.702	11.039	7.749	2.74%
Consistency between Content and Conclusion	0.707	10.6	7.496	2.65%
Sufficiency of Argumentation	0.734	10.171	7.463	2.64%
Internal Consistency of Research Findings	0.711	10.01	7.118	2.52%
Rationality of Argumentation	0.698	9.869	6.888	2.43%
Direct Correspondence between Conclusions and Results	0.712	9.614	6.849	2.42%
Consistency between Data Analysis and Conclusions	0.718	9.361	6.72	2.37%

The three highest-weighted indicators underscore a clear orientation toward impact and innovation. “Social significance” emerges as the most critical indicator (5.34%), signifying that the ecosystem’s long-term viability is deeply connected to its ability to address practical educational challenges and inform policy. This external relevance acts as a primary justification for the ecosystem’s existence and growth and ensures that its outputs remain valuable to society. The second-highest weight is assigned to “novelty of research methods” (4.29%), highlighting the system’s emphasis on adaptive capacity. Methodological innovation is a key driver for advancing scientific rigor and exploring new frontiers, preventing stagnation and ensuring the ecosystem can evolve. Closely following is the “accessibility of raw data” (4.21%), which points to the importance of transparency and reproducibility as pillars of a sustainable academic environment. Open data fosters a collaborative and self-correcting system, accelerating knowledge accumulation and reinforcing the collective credibility of the research community.

Conversely, indicators with lower weights, such as “consistency between data analysis and conclusions” (2.37%), are not unimportant but rather represent the foundational bedrock of the ecosystem. Their lower weights in the CRITIC analysis suggest they exhibit less variance across the sample, indicating that they are established “hygiene” factors that are largely standardized in published research. While these elements are essential for individual study credibility, they are less powerful in differentiating the overall developmental trajectory of the ecosystem compared to the dynamic drivers of social impact and innovation. Similarly, indicators like “transparency of analysis” (3.53%) and “alignment of data presentation with raw data” (3.41%) are crucial for ethical and scientific integrity, forming the essential scaffolding upon which more impactful and innovative research is built.

#### 3.1.3. Fuzzy comprehensive evaluation of ecosystem sustainability (2004–2023).

To synthesize the multi-dimensional data into a single, holistic assessment of the research ecosystem’s overall health and long-term sustainability, this study employs the fuzzy comprehensive evaluation method. This approach aggregates the performance across all 31 weighted indicators into a comprehensive sustainability index, providing a robust measure of the system’s developmental state. The comprehensive evaluation score S is calculated as the product of the objective weight vector W and the fuzzy relation matrix R:


S=W×R (6)

This operation provides the degrees of membership for 10 distinct quality levels, which identify the ecosystem’s character. The membership degrees for levels 1–10 are as follows: 0.0, 0.0, 0.0, 0.001, 0.011, 0.096, 0.328, 0.435, 0.124, and 0.004.

The distribution of these membership degrees reveals several key characteristics of the ecosystem’s sustainability. The complete absence of membership in the lowest quality tiers (levels 1 and 2) demonstrates that the ecosystem has successfully established a foundational standard of scholarly rigor, a critical prerequisite for sustainable development. The significant concentration of membership at level 7 (0.328) and level 8 (0.435), with a combined value exceeding 0.7, indicates that the ecosystem’s current state of health is robust and predominantly characterized by high-quality, effective research practices. This reflects a system that is not only functioning but maturing. Meanwhile, the tapering membership at the highest levels of excellence—level 9 (0.124) and level 10 (0.004)—highlights the remaining capacity for growth and the potential for enhancing the system’s future impact and adaptive capacity.

To quantify this overall state of health, the membership degrees were used to calculate a single composite score on a 100-point scale. The overall sustainability index for China’s empirical educational research ecosystem from 2004 to 2023 is 75.77. This score places the ecosystem’s performance in the upper quartile, signifying a resilient and well-developed system with a strong capacity for sustained, high-quality output.

**Fig 2:**
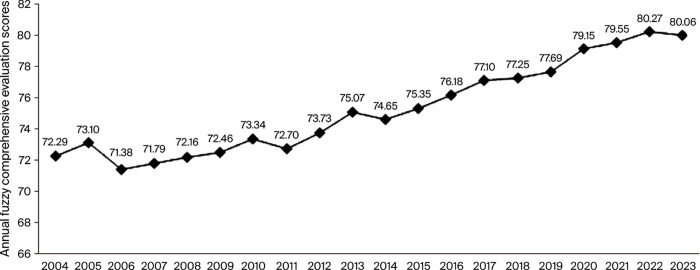
Annual evaluation results of empirical educational research journal articles in China from 2004 to 2023.

Furthermore, a longitudinal analysis of the year-by-year scores provides compelling evidence of the ecosystem’s positive evolutionary trajectory (Fig 2). To facilitate interpretation, the composite sustainability index is scaled from 0 to 100, where values below 60 indicate an emerging stage, scores between 60 and 80 reflect a developing-to-maturing stage, and scores above 80 denote a highly advanced stage of ecosystem development. Within this heuristic framework, the mean score of 75.77 suggests that China’s empirical educational research system has moved into the upper segment of the developing–maturing range: it is no longer in an early, fragile state but has not yet reached the level of systemic excellence implied by scores above 80.Although direct numerical comparisons across countries and disciplines are not possible with the present data, this interpretation is broadly consistent with cross-national scient metric studies and global analyses of higher education research output, which portray China as a rapidly expanding yet still consolidating contributor to international educational scholarship. Over the 20-year period, the sustainability score increased by eight points—a total growth of 10.75%. This demonstrates the system’s adaptive capacity and its ability to strengthen over time. The steady average annual growth rate of approximately 0.54% is indicative of a maturing system that has moved beyond initial volatility toward stable, incremental improvement, a hallmark of long-term sustainability.

### 3.2. Phase characteristics of chinese empirical educational research over the past twenty years

**Fig 3:**
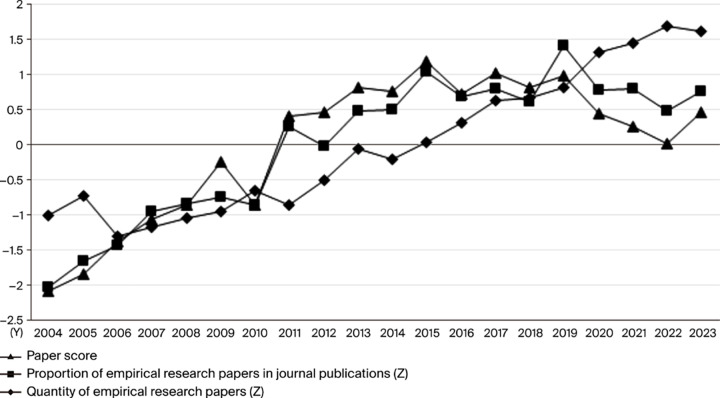
Development trends of empirical educational research in China from 2004 to 2023.

To understand the long-term sustainability of the research ecosystem, its developmental trajectory must be analyzed over time. Drawing from Derek de Solla Price’s theory of scientific development, research fields typically mature through distinct stages of initiation, growth, and stabilization (Price, 1963). This study applies this theoretical lens to assess the maturation of China’s empirical educational research ecosystem over the past two decades. By standardizing and analyzing key metrics—namely the annual volume of publications, the proportion of empirical articles, and the overall quality scores—we can map the ecosystem’s evolution. This longitudinal analysis reveals a clear progression through three phases, reflecting the ecosystem’s journey from emergence and volatility to rapid consolidation and, finally, to a state of sustained, maturing improvement (Fig 3). This phased development mirrors the general patterns of how complex research systems gain stability, resilience, and capacity over time.

#### 3.2.1. 2004–2010: Fluctuating development phase.

The period between 2004 and 2010 marks the ecosystem’s nascent and exploratory stage, characterized by the inherent volatility of a system in its infancy. During these formative years, the ecosystem’s outputs were inconsistent, with significant fluctuations in both the quantity and quality of research. This instability was driven by two primary foundational constraints: limited academic resources and underdeveloped methodological capacity. Researchers often displayed a superficial grasp of empirical methods, leading to non-standardized approaches, a reliance on basic statistical descriptions, and low data transparency, which are common challenges for an emerging research ecosystem. Consequently, the system’s focus was largely internal, concentrating on experiential accounts and theoretical groundwork with minimal direct policy application or interdisciplinary integration.

The quantitative data clearly illustrates this phase’s instability. Year-by-year fuzzy comprehensive evaluation scores hovered around 72 between 2004 and 2006, reflecting a system struggling to establish a consistent quality baseline. However, crucial external inputs began to catalyze development. The Ministry of Education of the People’s Republic of China’s 2006 policy(*Ministry of Education’s Opinions on Vigorously Improving the Quality of Philosophical and Social Sciences Research in Higher Education Institutions*) explicitly calling to “emphasize empirical studies” provided a critical injection of legitimacy and direction. This was followed by the landmark *National Medium- and Long-Term Education Reform and Development Plan Outline (2010–2020)*, which further solidified the demand for evidence-based research. These policy drivers acted as powerful catalysts, signaling the strategic importance of this research area and prompting the ecosystem to mature. In response, the quality score began a noticeable increase after 2007, reaching a peak of 73.34 in 2010, marking the first tangible sign of the ecosystem’s stabilization and its transition toward a more sustainable growth path.

#### 3.2.2. 2011–2015: Rapid growth phase.

Following its foundational stage, the research ecosystem entered a period of accelerated development between 2011 and 2015, transitioning from instability to robust growth. This phase was characterized by a fundamental strengthening of the ecosystem’s internal structures and its external connectivity, laying the groundwork for long-term sustainability.

A key development was the ecosystem’s increased responsiveness to the national context, with many studies actively aligning with educational policy reforms and fostering direct interaction between scholars, administrative departments, and schools. This created crucial feedback loops that enhanced the practical application and impact of research findings. Concurrently, the ecosystem’s intellectual foundations broadened as interdisciplinary integration with fields like public policy, sociology, and psychology became a prominent trend, deepening theoretical insights and enhancing the system’s adaptive capacity.

This period saw a significant expansion in both the quantity and quality of empirical research, with concepts like “data reproducibility” and “research verifiability” gaining widespread acceptance within the academic community. The ecosystem’s improving health is quantitatively evident in the steady rise in its quality scores, which climbed from 73.7 to 75.35 over these five years. A notable leap occurred between 2012 and 2013, when the score jumped from 73.73 to 75.07, signaling that the system had reached a new threshold of maturity and quality. This rapid maturation was propelled by two synergistic forces.

On the one hand, sustained national policy support acted as a powerful external catalyst. Government initiatives prioritizing educational quality, such as the *National Ethnic Education Research Plan (2014–2020)*, with its explicit call for “strengthening evidence-based policy research”, provided the strategic direction and resources necessary to fuel the ecosystem’s growth.

On the other hand, the ecosystem began to develop its own internal momentum through the self-organization of academic forces. The establishment of robust platforms for scholarly exchange, such as the inaugural National Forum on Empirical Educational Research in 2015 and a thematic conference by the Educational Theory Journals Branch the same year, were instrumental. These forums solidified a community of practice, accelerated knowledge dissemination, and reinforced emerging norms, creating the internal infrastructure vital for a resilient and sustainable research ecosystem.

#### 3.2.3. 2016–2023: Continuous improvement phase.

Entering its most recent stage, the research ecosystem transitioned from rapid growth to a phase of sustained maturation and refinement, demonstrating the characteristics of a stable and resilient system. The overall quality of empirical research further improved, with a heightened emphasis on rigorous theoretical frameworks and the explication of causal mechanisms. This period was marked by the system’s enhanced adaptive capacity, as it began to integrate new tools and methods, particularly big data, and absorb influences from adjacent scientific fields like cognitive neuroscience and information science, which broadened the paradigms and boundaries of educational inquiry. Methodologically, this maturity manifested in the increased prevalence of sophisticated hybrid and interdisciplinary approaches. Key indicators of the ecosystem’s health included the widespread adoption of standardized empirical methods, leading to greater reliability and validity of findings, and a deeper level of interdisciplinary integration that moved beyond a single-discipline focus.

Quantitatively, the ecosystem’s outputs remained at a high and stable level, with standardized metrics for publication volume and quality scores consistently in the positive range. Notably, the temporary decline in publication volume from 2020 to 2022, which this study attributes to data collection challenges during the global public health crisis, was followed by a recovery in 2023. This pattern provides evidence of the ecosystem’s resilience—its ability to absorb external shocks and return to a productive state. Most significantly, the fuzzy comprehensive evaluation scores showed a consistent upward trajectory, approaching or exceeding 80 after 2020 and reaching a peak of 80.27 in 2022, indicating that the system’s quality benchmark had reached a new, higher plateau.

This sustained improvement was fueled by a virtuous cycle of national policy support and proactive self-organization by the academic community. For instance, the 2017 “National Joint Conference on Empirical Educational Research” and its resulting “Action Declaration” signaled the community’s commitment to self-regulation and quality enhancement. This internal momentum was reinforced by external policy drivers, including the Ministry of Education of the People’s Republic of China’s 2019 call to “enhance empirical research”(*Opinions on Strengthening Educational Scientific Research in the New Era*) and its 2020 mandate requiring key laboratories to demonstrate a “distinctive focus on empirical research.”

During this mature phase, China’s empirical educational research ecosystem not only solidified its domestic influence but also established a notable presence within the international academic community. The challenges now facing the system—such as maintaining global competitiveness and continuing to advance data quality and methodological innovation—are indicative of a mature ecosystem focused on pushing the frontiers of knowledge rather than merely establishing its foundations.

## 4. Discussion

This study employed a generative AI-based methodology to conduct a longitudinal sustainability assessment of China’s empirical educational research ecosystem from 2004 to 2023. The findings reveal a complex system characterized by a positive macro-level trajectory toward maturity and resilience while also marked by persistent micro-level vulnerabilities that require targeted intervention. This discussion interprets these findings, explores their broader implications for academic governance, acknowledges the study’s limitations, and proposes directions for future research.

### 4.1. Interpretation of findings: A maturing ecosystem at a crossroads

The comprehensive evaluation score of 75.77 and the steady eight-point increase over two decades paint a picture of an ecosystem that has successfully navigated its formative stages and is progressing toward sustainable, high-quality development. The evolutionary path, clearly demarcated into phases of fluctuating development (2004–2010), rapid growth (2011–2015), and continuous improvement (2016–2023), aligns with classic theories of scientific maturation and demonstrates the system’s capacity to evolve from instability to a state of robust and resilient output. This progression was not accidental but was propelled by a synergistic interplay between external policy catalysts, such as government mandates for evidence-based research, and the internal self-organization of the academic community through scholarly forums and emergent norms.

However, the ecosystem’s health is not uniform. A significant paradox emerges from the analysis: the system’s greatest strength may also be linked to its primary weaknesses. The highest-scoring indicators were “relevance of research problem” and “social significance”, reflecting a research agenda that is highly responsive to national policy priorities and practical educational challenges. This strong external orientation, while ensuring the ecosystem’s societal value, may inadvertently divert focus from foundational scientific practices. The comparatively low scores for “accessibility of raw data” and “transparency of data analysis” point to a systemic deficit in open science.

This paradox is not merely a technical imbalance but a reflection of deep-seated institutional and cultural dynamics. Institutionally, the evaluation system for researchers in China has long prioritized rapid publication in response to policy shifts, fostering a “responsiveness” culture. Culturally, this has created a pragmatic approach to research where aligning with national strategy guarantees resources and recognition. However, this same mechanism can disincentivize the high-risk, time-consuming efforts required for methodological innovation and data transparency, as these foundational practices often yield slower returns in the current evaluation framework. Thus, the system excels at “answering” policy questions but struggles with “opening” the scientific process.

These system dynamics align with global discourse in the “Science of Science”, specifically regarding the tension between societal relevance and methodological rigor. The paradox observed in China’s ecosystem—high relevance but lower transparency—mirrors the “reproducibility crisis” noted in Western psychology and social sciences. Theories of scientific evolution suggest that emerging ecosystems often prioritize rapid expansion and application before stabilizing into rigorous, verified disciplinary structures. Therefore, the identified lag in data transparency is not merely a local deficit but a characteristic symptom of an ecosystem transitioning from a “growth-first” to a “quality-first” paradigm, requiring similar open-science interventions as seen internationally.

These dynamics suggest that China’s empirical educational research ecosystem is undergoing a transition that moving from expansionary, policy-driven growth toward a quality-first, open-science paradigm. This positions the Chinese case as a valuable empirical lens for theorizing how national research ecosystems worldwide can navigate the trade-offs between societal responsiveness and methodological robustness.

### 4.2. Implications for theory and practice

Building on the above interpretation of China’s research ecosystem as a system in transition—from policy-driven expansion toward a quality-first, open-science paradigm—we now turn to its broader theoretical and practical implications.

The primary theoretical contribution of this study is the operationalization of “research ecosystem sustainability” as a measurable construct. By moving beyond traditional bibliometrics to a nuanced, multi-indicator qualitative assessment performed at scale, this work provides a new conceptual and methodological framework for metascience and academic evaluation. It demonstrates that generative AI can serve not only as an object of study but as a powerful analytical tool for assessing the health and trajectory of complex social systems.

Practically, the findings offer a data-driven diagnostic for policymakers and academic administrators. The clear identification of strengths and weaknesses provides a roadmap for targeted interventions. Future policy should aim to build upon the ecosystem’s established relevance by creating robust data infrastructure and incentivizing open research practices. For journal editors and university governors, the results underscore the need to enforce more rigorous standards for data sharing and to actively foster a culture that values methodological innovation alongside topical relevance. From an international perspective, situating the Chinese case within the global agenda of SDG 4 suggests that the dynamics observed here are not idiosyncratic, but illustrative of the challenges faced by many rapidly developing research systems. Our GAI-based framework can therefore serve as a transferable diagnostic tool to support comparative cross-national monitoring, mutual policy learning, and the co-construction of more equitable and resilient global research ecosystems.

### 4.3. Limitations and future directions

Artificial intelligence in education tools significantly contribute to innovation, broadened educational access, and sustainability across the society, economy, and environment pillars [36]. Despite the robustness of the methodology, several limitations must be acknowledged. First, the GAI evaluation, while validated against expert scores and multiple models, operates as a “black box” to some extent; its scoring may be influenced by latent biases within its training data. Second, our sample, while representative of high-level research in China, was drawn from four leading domestic journals. This focus necessarily excludes research published in international venues, books, and other outlets, meaning our findings are representative of the core of the domestic ecosystem but may not be generalizable to its entire intellectual output. Third, the evaluation framework itself, though comprehensive, is a constructed representation of research quality and omits certain dimensions, like academic ethics, which were presumed to have been handled by the journals’ pre-publication processes.

Furthermore, the integration of Generative AI into research evaluation introduces critical ethical and epistemological challenges. While AI offers unprecedented scalability, it is not immune to algorithmic bias, potentially reproducing historical inequities present in its training data. Epistemologically, we must remain vigilant against “metric fixation”, ensuring that AI-generated scores function as a heuristic aid for human experts rather than a definitive judgment. Future governance of AI in bibliometrics must prioritize fairness and accountability, establishing “human-in-the-loop” protocols to validate AI assessments against community consensus.

These limitations open several avenues for future research. A crucial next step would be to apply this GAI-based framework in comparative analyses, assessing the developmental trajectories of educational research ecosystems in other countries or different academic disciplines within China. Further research could also focus on refining the evaluation tool by fine-tuning the large language model on expert-annotated papers to enhance the nuance of its assessments. Finally, this study provides a 20-year baseline; its true value will be realized through continued longitudinal tracking, allowing for the real-time monitoring of the ecosystem’s health and the assessment of future policy interventions designed to remedy the weaknesses identified herein.

## 5. Conclusions and recommendations

### 5.1. Conclusions

This study used a generative AI-driven methodology to conduct a 20-year longitudinal sustainability assessment of China’s empirical educational research ecosystem (2004–2023) (2004–2023). The results depict an ecosystem that has moved through three distinct phases—fluctuating development, rapid growth, and continuous improvement—toward a new plateau of stable, high-quality output, as reflected in the steady rise of the comprehensive sustainability index to 75.77. At the same time, our micro-level diagnosis reveals a defining paradox: the system’s strong policy alignment and problem relevance coexist with persistent weaknesses in data transparency and methodological innovation, indicating that the ecosystem is at a pivotal juncture in its transition from a policy-driven to a quality-first, open-science paradigm.

However, a micro-level diagnosis uncovers a critical paradox that defines the ecosystem’s current state. While it excels in areas of high societal and practical relevance, demonstrating a strong capacity to address real-world educational problems, it exhibits persistent vulnerabilities in foundational scientific practices. Specifically, deficiencies in data transparency, accessibility of raw data, and methodological innovation pose significant challenges to its long-term credibility, reproducibility, and global competitiveness. China’s empirical educational research ecosystem has reached a pivotal juncture where it must transition from a system primarily driven by external policy demands to one that also prioritizes the internal scaffolding of open science and methodological rigor. Achieving this balance is the central task required in order to ensure its future as a resilient, innovative, and globally integrated system. By explicitly characterizing this phased evolution and its underlying drivers, our study offers a dynamic systems perspective on how national research ecosystems can be steered toward long-term sustainability rather than short-term, policy-cycled fluctuations.

### 5.2. Recommendations

Building on the comprehensive evaluation, this study proposes four targeted strategies to address the identified weaknesses and foster the sustainable development of China’s empirical educational research ecosystem. These strategies also offer transferable design principles for other countries seeking to align educational research with the long-term objectives of SDG 4 (Quality Education).

Firstly, we propose the construction of a tiered National Educational Research Data Infrastructure (NERDI) guided by FAIR principles (Findable, Accessible, Interoperable, Reusable). To directly address the identified deficits in data transparency (Score: 7.08), this platform should implement a “graded access mechanism”: Level 1 for fully open public data, and Level 2 for sensitive data accessible only to accredited researchers within secure enclaves. This specific strategy balances the urgent need for reproducibility with privacy concerns, directly remedying the ecosystem’s structural weakness in data availability.

Secondly, interdisciplinary reconstruction and methodological innovation should be fostered. The ecosystem’s long-term adaptive capacity depends on its ability to move beyond disciplinary fragmentation. This requires actively promoting collaborative networks that span education, computer science, sociology, and other fields to address complex problems. Furthermore, academic evaluation mechanisms must be reformed to recognize and reward interdisciplinary contributions and methodological novelty, thereby incentivizing researchers to expand their toolkits and address the identified gaps in innovation.

Thirdly, the translation of research to policy and practice should be systematized. Building on the ecosystem’s strength in addressing relevant problems, the pathways from research to impact must be formalized. This can be achieved by establishing AI-powered systems to intelligently match empirical findings with policy agendas and by creating embedded “research–practice partnerships” to facilitate the co-design and iterative testing of educational interventions in real-world settings.

Last but by no means least, an AI-augmented for ecosystem governance should be implemented. To ensure continuous improvement and resilience, the governance of the research ecosystem itself should be optimized. This includes introducing AI-augmented academic evaluation systems to enhance rigor and efficiency, as well as developing dynamic evaluation frameworks that use key metrics to monitor ecosystem health in real time. Adopting these intelligent tools will enable a more proactive and evidence-based approach to academic governance, reinforcing research integrity and sustaining the ecosystem’s positive developmental trajectory.

## Supporting information

S1 Table Scores of papers on metric. (XLSX)
